# Supplementary material for: Atlantic origin of the increasing Asian westerly jet interannual variability
Source: Nat Commun. 2024 Mar 9;15:2155. doi: 10.1038/s41467-024-46543-x (PMC10925044; doi:10.1038/s41467-024-46543-x)
Supplement: Supplementary file 1 — Supplementary Information [file 41467_2024_46543_MOESM1_ESM.pdf]

# Supplementary Information for

## Atlantic origin of the increasing Asian westerly jet interannual variability

Lifei Lin[1,2]*, Chundi Hu[1]*†, Bin Wang[3], Renguang Wu[4], Zeming Wu[1], Song Yang[2,5], Wenju Cai[6], Peiliang Li[1], Xuejun Xiong[1] & Dake Chen[2,7]

[1] Ocean College, Zhejiang University, Zhoushan, China

[2] School of Atmospheric Sciences, Sun Yat-sen University, & Southern Marine Science and Engineering Guangzhou Laboratory, Zhuhai, China

[3] Department of Atmospheric Sciences and International Pacific Research Center, University of Hawaii at Manoa, Honolulu, USA

[4] School of Earth Sciences, Zhejiang University, Hangzhou, China

[5] Guangdong Province Key Laboratory for Climate Change and Natural Disaster Studies, Sun Yat-sen University, Zhuhai, China

[6] Centre for Southern Hemisphere Oceans Research (CSHOR), CSIRO Oceans and Atmosphere, Hobart, TAS, Australia

[7] State Key Laboratory of Satellite Ocean Environment Dynamics, Second Institute of Oceanography, Ministry of Natural Resources, Hangzhou, China

**Contents:**

Supplementary Figures 1–13.

Supplementary Tables 1–5.

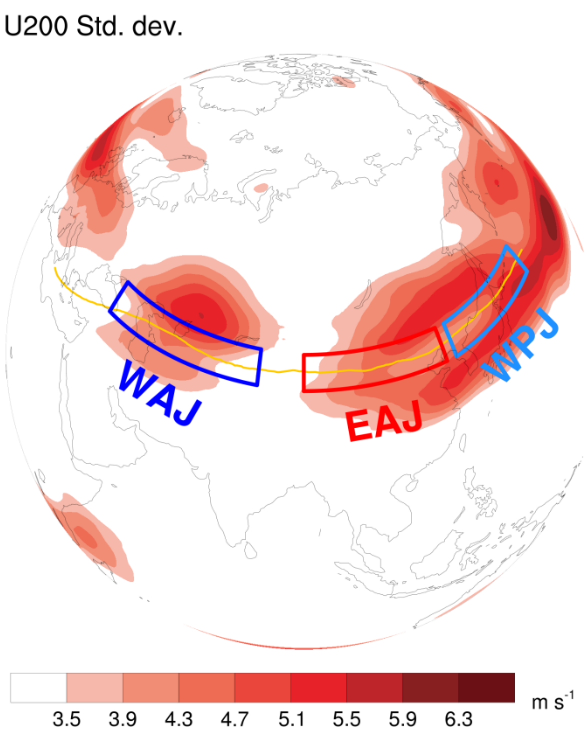


**Supplementary Fig. 1 | Interannual variability of Eurasian jet stream measured by the SD of U200** (units: m s$^{-1}$)**.** Three boxes denote definition regions of WAJ, EAJ and WPJ, same as in **Fig.1a**. Yellow line outlines the Eurasian jet axis at 200 hPa in high summer (July–August).

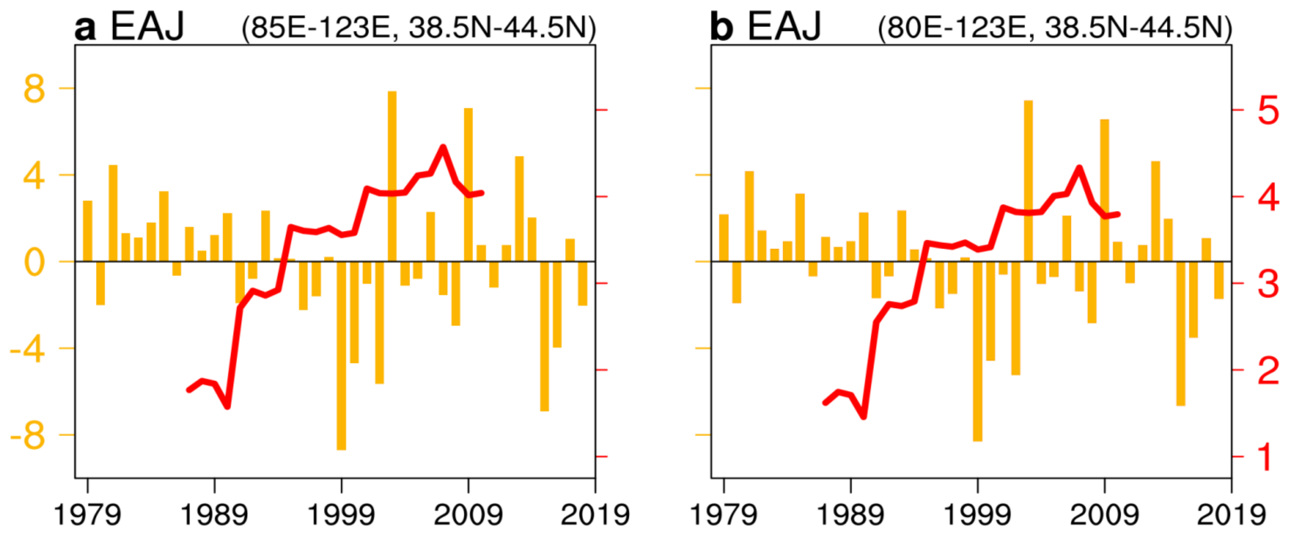


**Supplementary Fig. 2 | Sensitivity test of the interannual variability of EAJ index due to definition region.** Shown in each sub-figure is the year-to-year variations (yellow bars) and the corresponding 17-yr running SD (red curves; units: m s$^{-1}$) of EAJ index. Definition regions are shown in titles of each sub-figure.

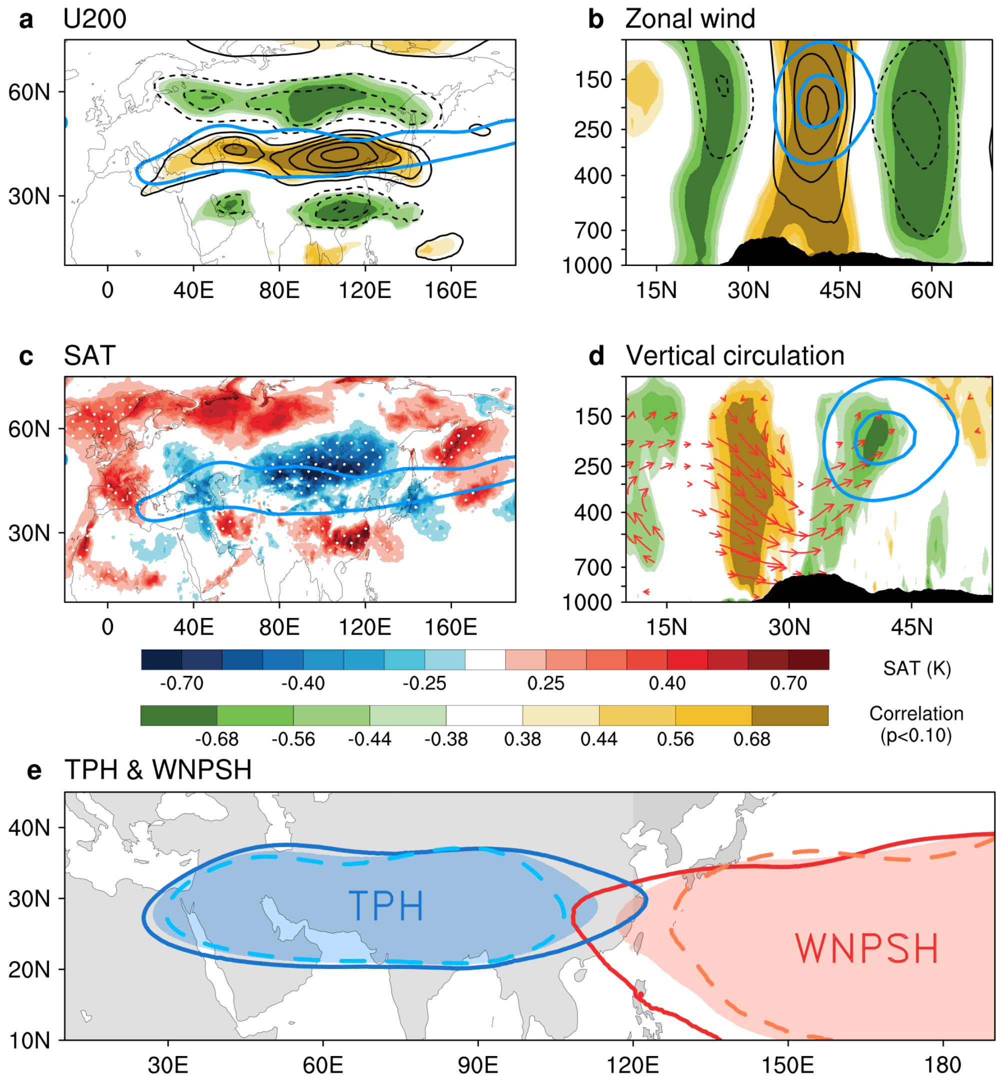


**Supplementary Fig. 3 | Circulation anomalies related to EAJ index during the post-1999 period.** **a**, U200 anomalies linked to the normalized EAJ index. Shading and black contour represent correlation coefficient and regression coefficient (units: m $s^{-1}$). The contour interval for regression maps is 1 m $s^{-1}$, with zero line omitted. Blue contours indicate the climatology for U200 in 20 m $s^{-1}$. **b**, Same as **a** except for the vertical-horizontal cross section of zonal wind anomalies averaged from 80°–130°E. The blue contours indicate the climatology for zonal wind in 15 and 25 m $s^{-1}$. **c**, Regression maps of SAT against the normalized EAJ index, with 90% significance stippled. **d**, Correlation (shading) fields of vertical wind anomalies and regressed wind (vector) in meridional section averaged from 80°–130°E with normalized EAJ index. The vertical wind is multiplied by 500 before drawing the vector. **e**, Composite of Tibet Plateau High (TPH) and western North Pacific subtropical high (WNPSH). Blue and red shadings represent the climatology of TPH (measured by H150 in 14350 gpm) and WNPSH (measured by H500 in 5865 gpm), respectively. Thick red (blue)

solid line represents the mean state of TPH (WNPSH) in strong EAJ years, while dash line represents that in weak EAJ years.

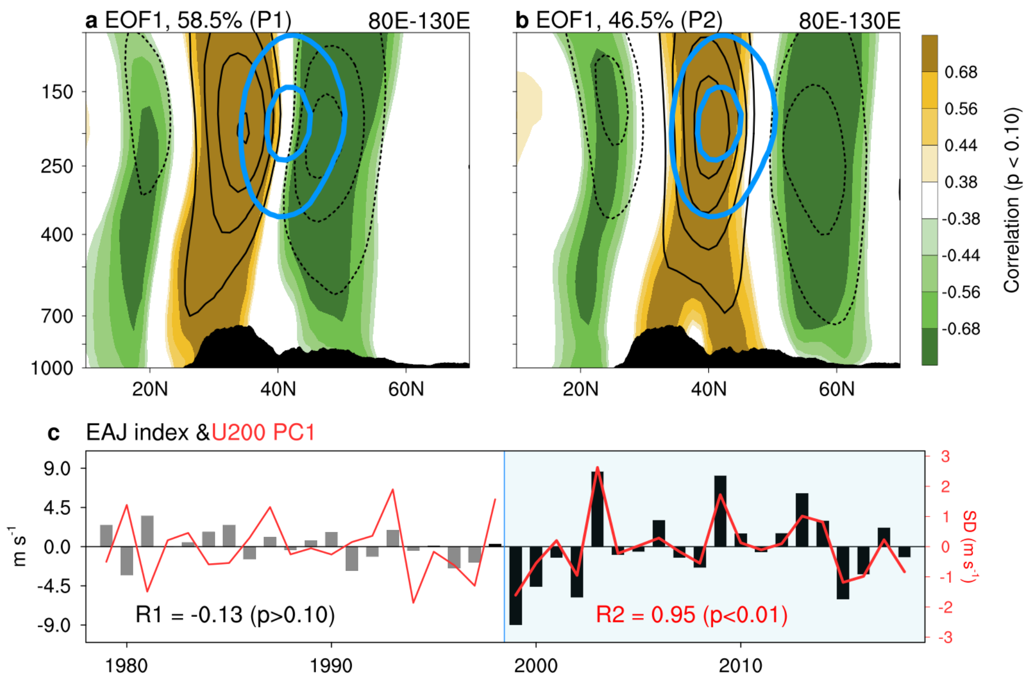


**Supplementary Fig. 4 | Shifting of leading mode of the zonal wind in vertical profile.** Same as **Fig. 3a, b, and e** but the EOF analysis is preformed using the meridional section of zonal wind (i.e., 15°–65°N, 1000-hPa–100-hPa; averaged from 80°E to 130°E). The blue contours indicate the climatology for zonal wind in 15 and 25 m $s^{-1}$.

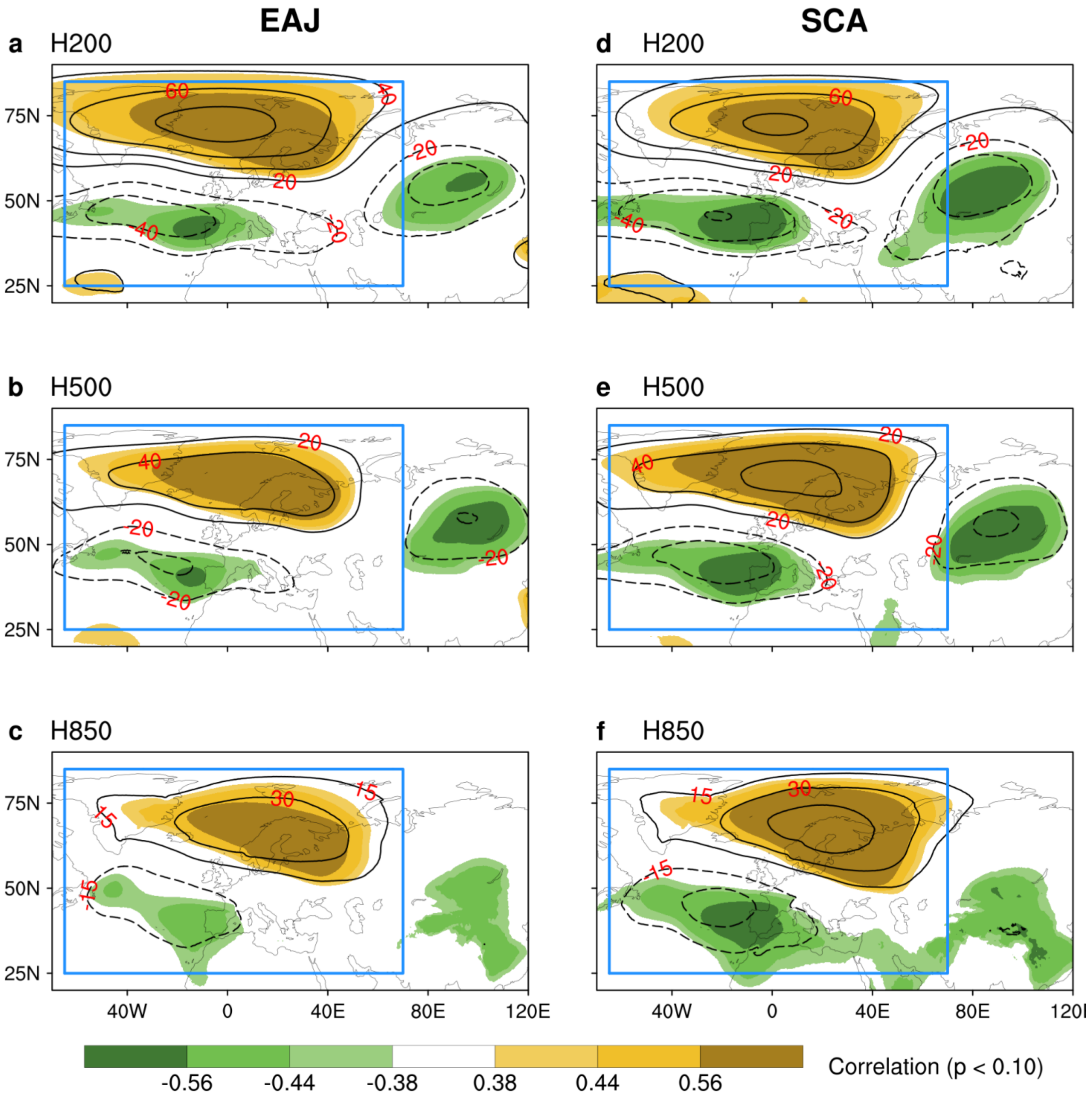


**Supplementary Fig. 5 | Comparing circulation patterns of EAJ and SCA at February. a–c**, Correlation (shading) and Regression maps (contour; units: gpm) of (**a**) H200, (**b**) H500 and (**c**) H850 in February associated with EAJ index for the post-1999 period. **d–f**, Same as **a–c**, except for the February SCA index. Blue boxes outline the same region (25°N–85°N, 65°W–70°E) used for calculating their pattern correlations, as shown in the following **Supplementary Table 4**.

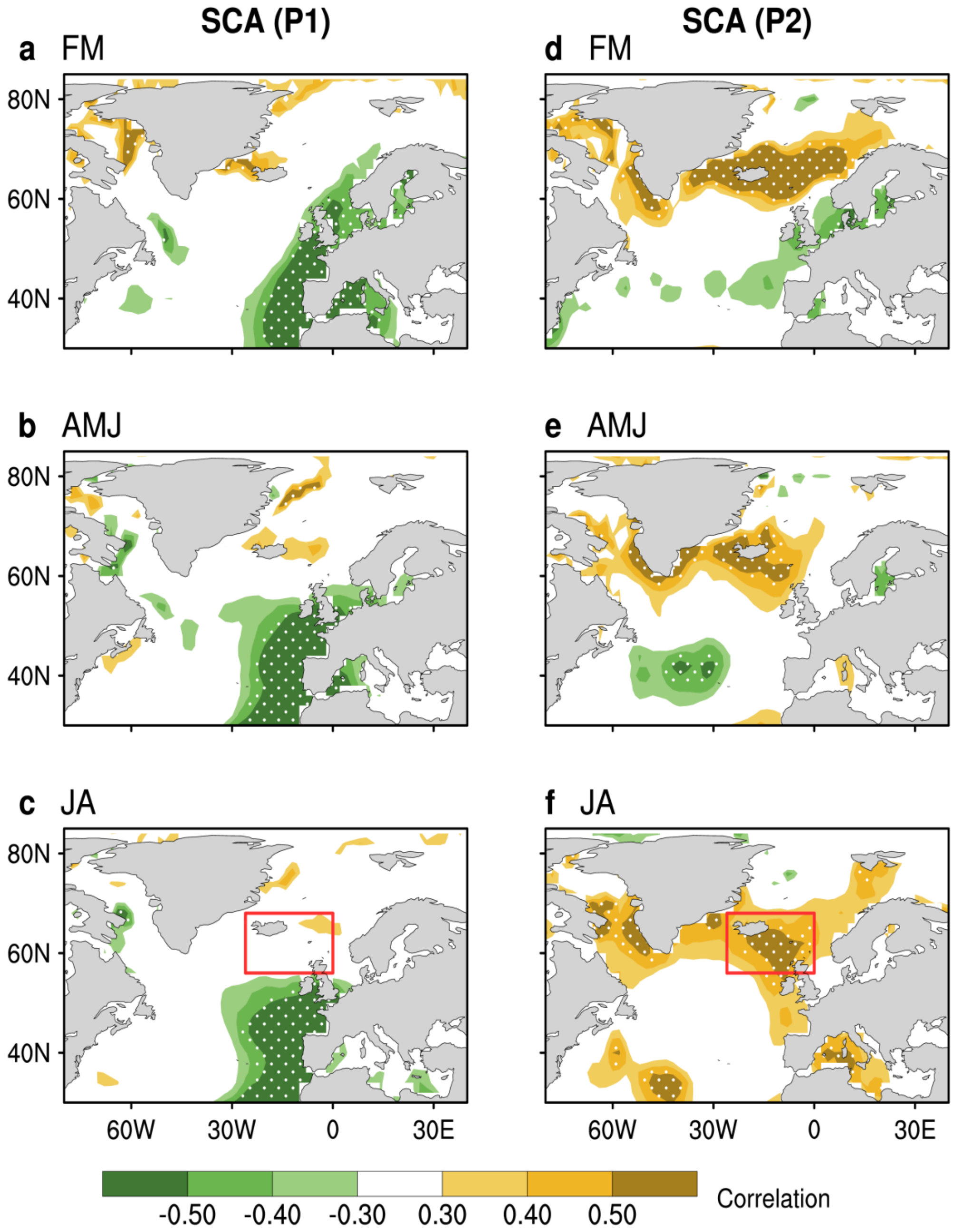


**Supplementary Fig. 6 | Distinct impacts of February SCA pattern on SST for two sub-periods.** **a–c**, Correlation maps of SST anomalies with the SCA index during the pre-1998 period, at February–March (**a**), April–June (**b**) and July–August (**c**). **d–f**, Same as **a–c** but for the post-1999 period. Dotted areas are statically at the 0.05 level. The red box outlines the key region 56°N–68°N/26°W–0°.

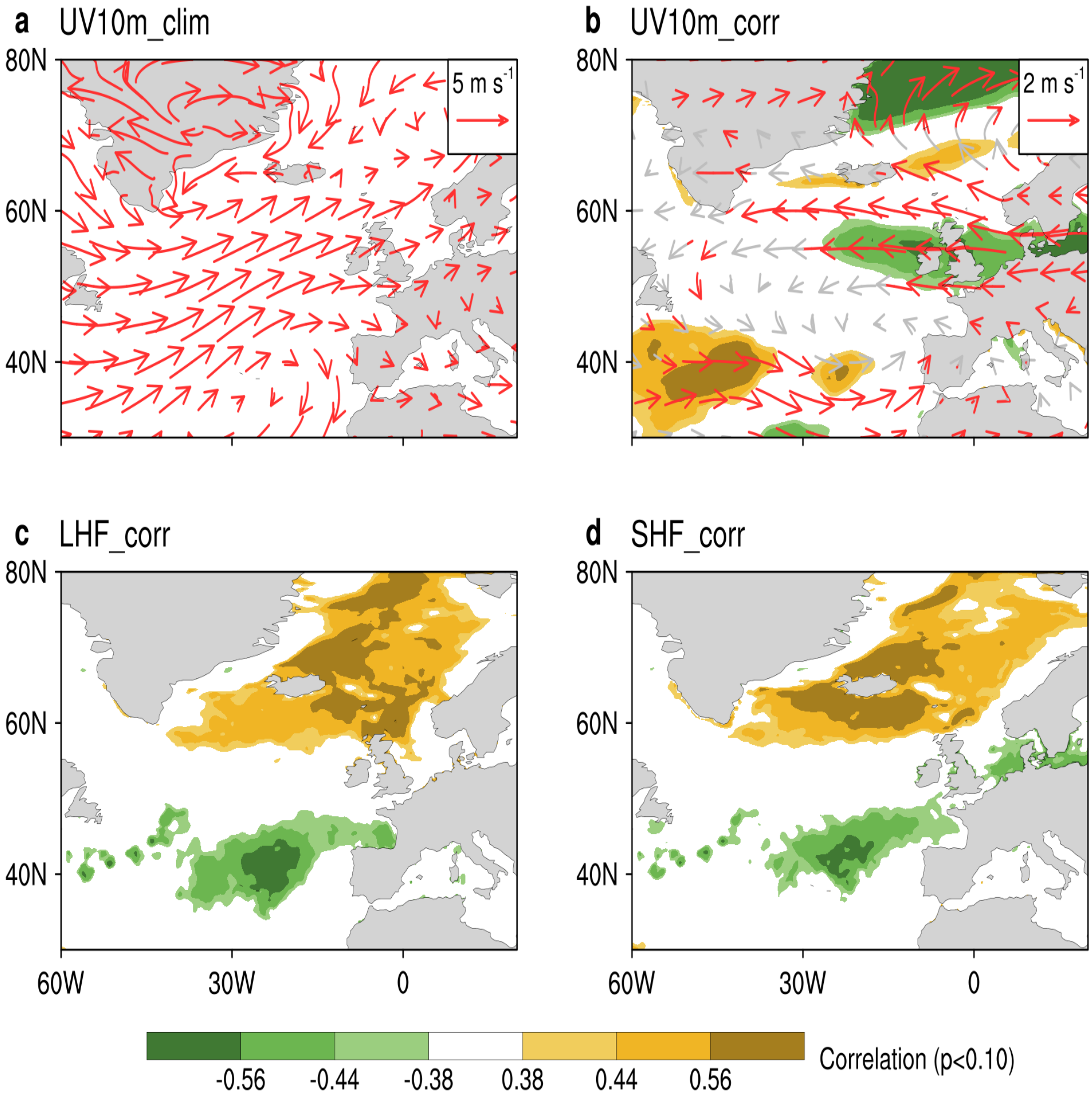


**Supplementary Fig. 7 | Circulation anomalies related to SCA index during the post-1999 period. a**, Climatology of Surface wind (UV10m; unit: m s$^{-1}$). **b–d**, Correlation maps of horizontal wind speed (**b**), LHF (**c**) and SHF (**d**) with SCA index. Correlation coefficients above 90% confidence level are colored. Red boxes denote regions for computing pattern correlation. Vectors in (**b**) represents the regressed UV10m against the normalized SCA index, with 90% significance colored in red.

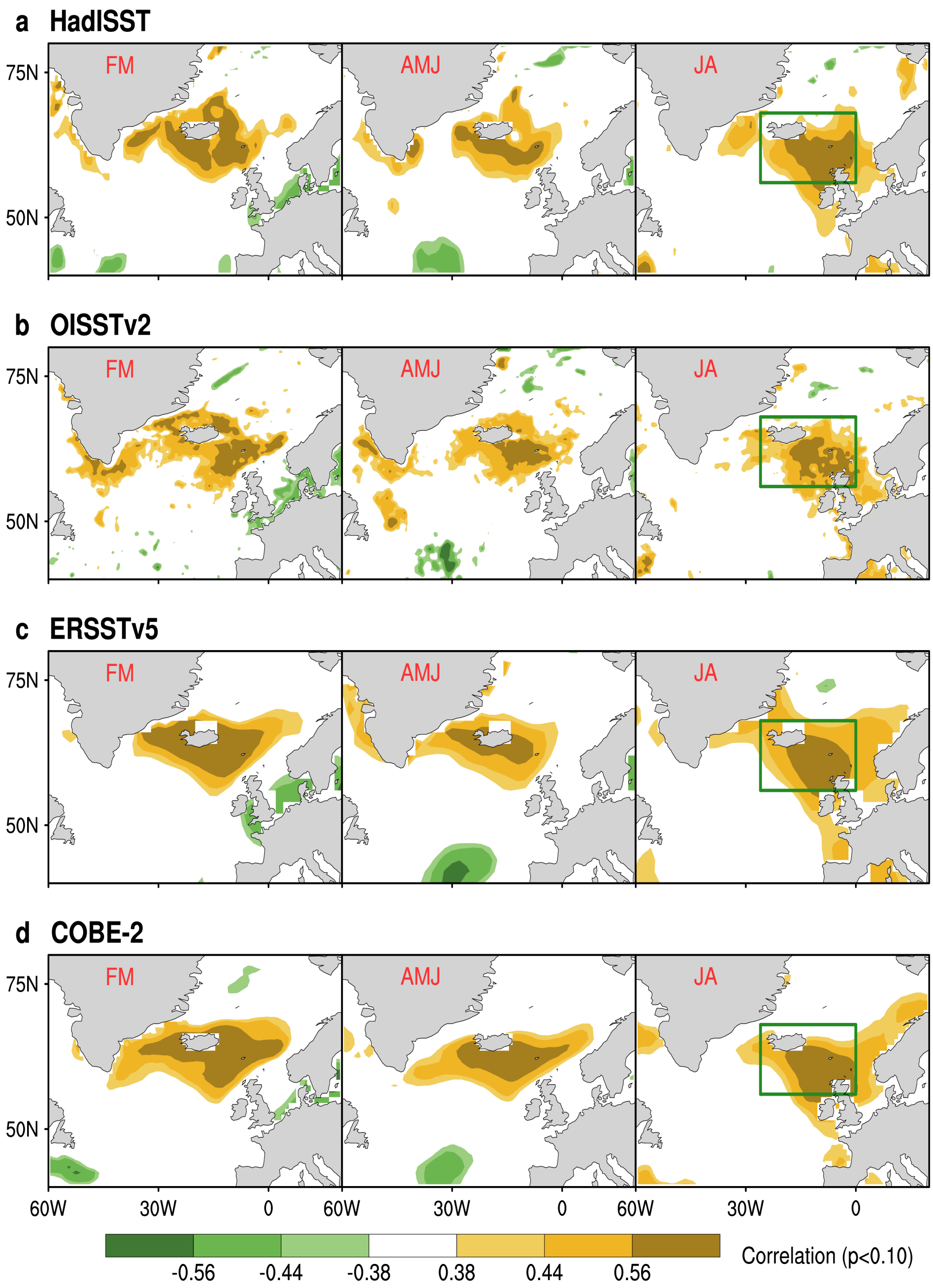


**Supplementary Fig. 8 | SST anomalies in multi-data related to EAJ index during the post-1999 period. a–d**, Correlation maps of SST anomalies with the EAJ index from preceding February to simultaneous summer, using multi-source SST reanalysis datasets including HadISST (**a**), OISSTv2

(**b**), ERSSTv5 (**c**) and COBE-SST2 (**d**). The green box outlines the key region 56°N–68°N/26°W–0°.

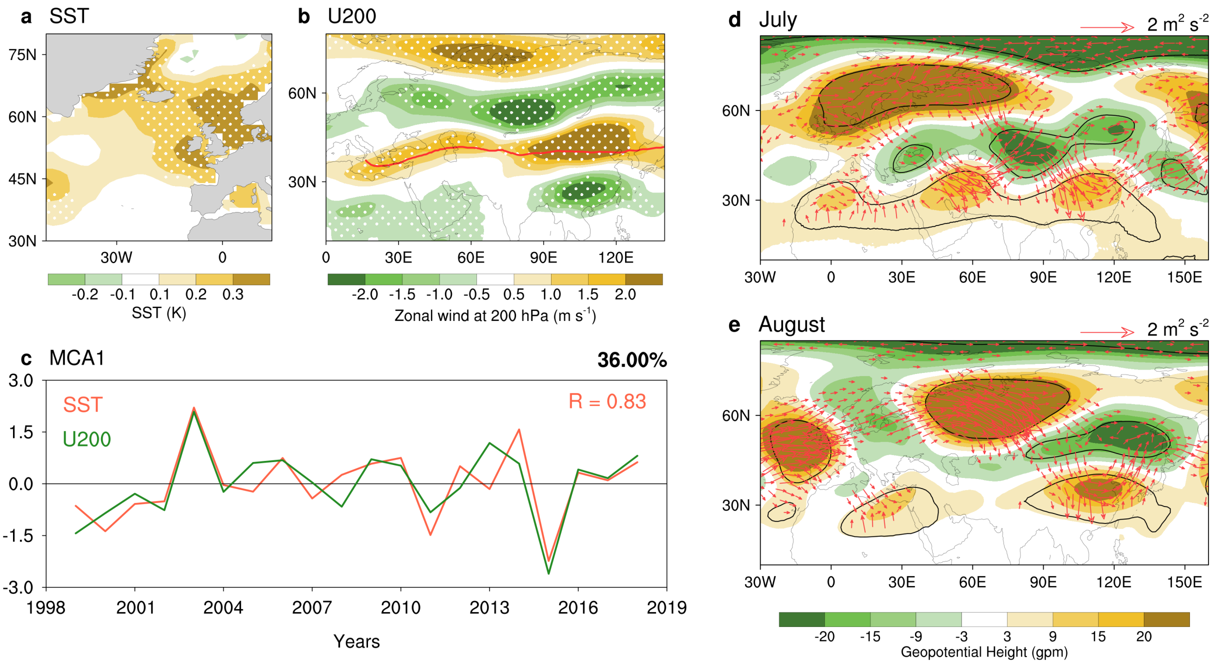


**Supplementary Fig. 9 | The first leading MCA mode (MCA1) of co-varying North Atlantic SST and Eurasian circulation (U200) in high summer. a–b**, the heterogeneous regression (shading; units: K in a and m $s^{-1}$ in b) maps against the temporal coefficients corresponding to MCA1, with 90% significance stippled. The red line in (**b**) indicates the jet axis. **c**, Time series of MCA1-SST (red) and MCA1-U200 (green). **d–e**, Regression maps of 300-hPa geopotential height (shading; units: gpm) onto the normalized MCA1-U200 time series in (**d**) July and (**e**) August, with significant region ($p < 0.10$) enclosed by black line. Overlapped vectors represent the corresponding Wave Activity Flux (units: $m^2$ $s^{-2}$; see Methods) at 300 hPa.

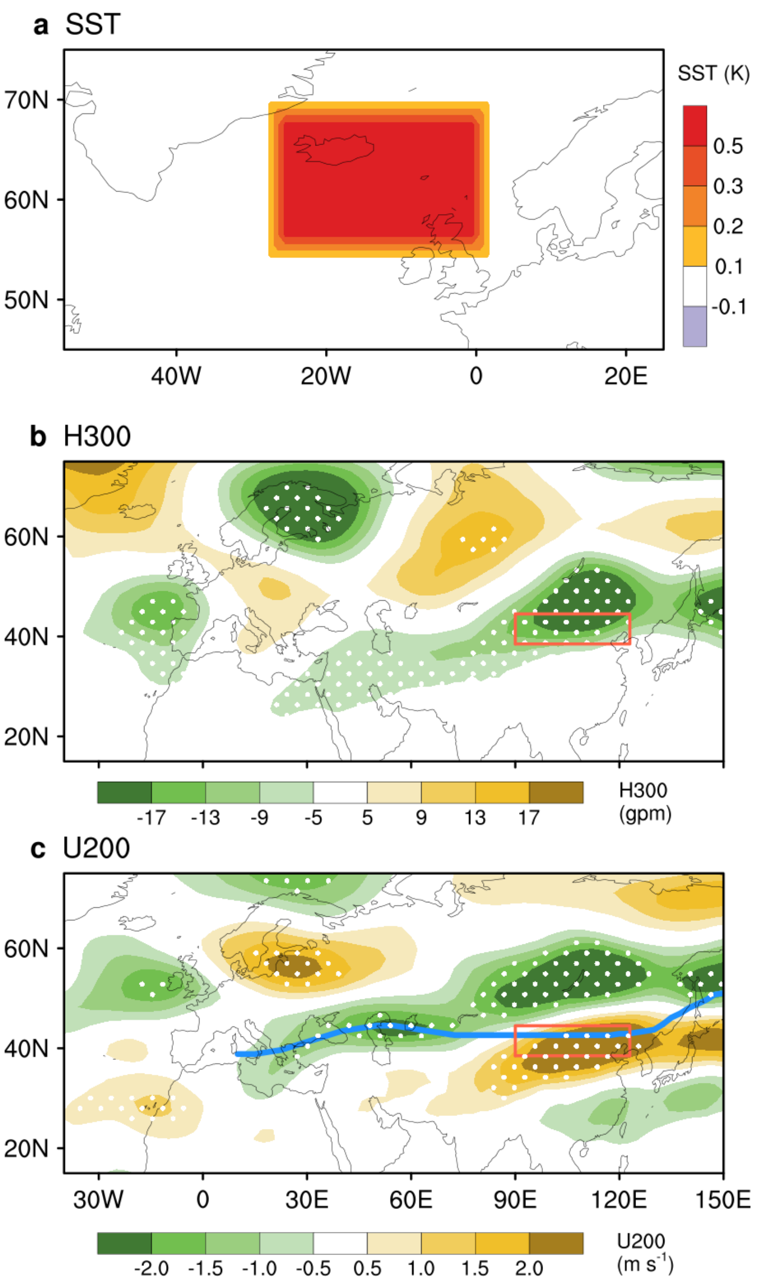


**Supplementary Fig. 10 | Simulated atmospheric response to SST forcing. a**, High summer (JA) SST anomalies (shading; units: K) near the Iceland as the oceanic forcing in CAM4 experiment. **b–c**, Simulated atmospheric response in (**b**) H300 and (**c**) U200 to prescribed oceanic forcing with 95% significance stippled. The red box outlines the region used for defining the EAJ index. The blue line in (**c**) indicates the jet axis.

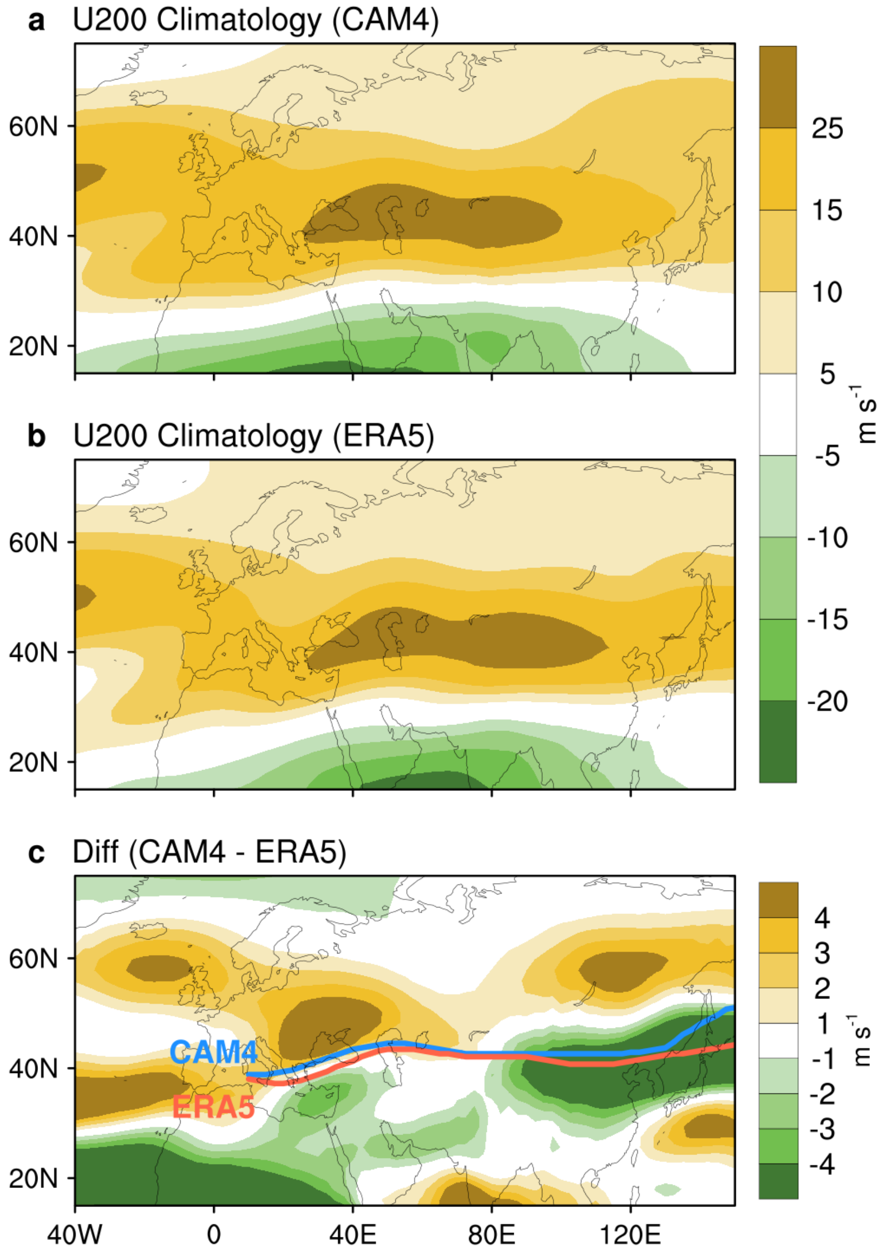


**Supplementary Fig. 11 | Mean state bias of CAM4 model. a**, the mean state of U200 (units: m $s^{-1}$) in CAM4 model in CTRL run as the climatology of model. **b**, Climatology of U200 (units: m $s^{-1}$) during 1979-2018, obtained from ERA5 data. **c**, Difference between climatology of CAM4 and ERA5, with the corresponding jet stream axis (blue for CAM4 and red for ERA5).

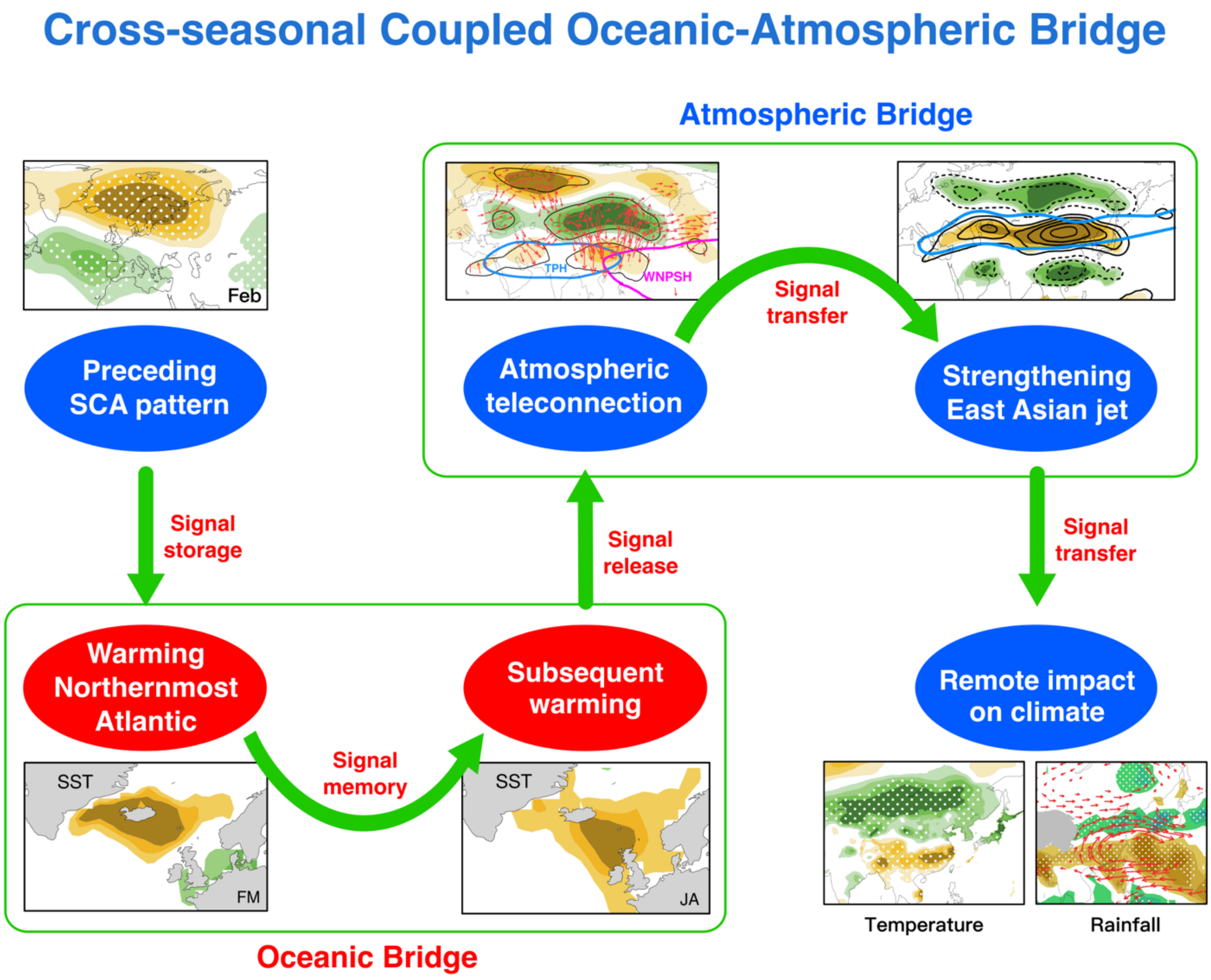


**Supplementary Fig. 12 | Schematic diagram illustrating the concept of "Coupled Oceanic-Atmospheric Bridge".** This bridge linking preceding SCA pattern over the North Atlantic and ensuing summer EAJ: SCA pattern in positive phase can lead to strengthening EAJ.

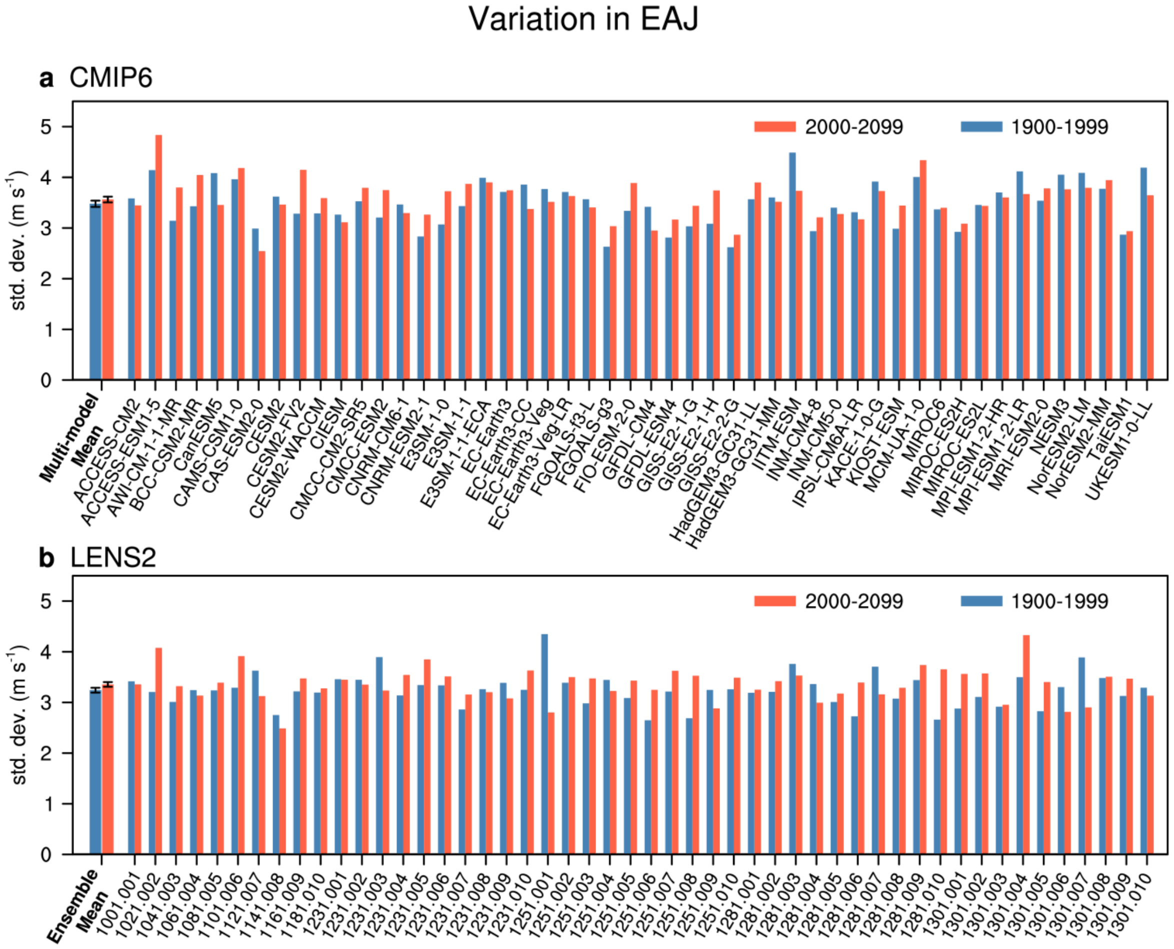


**Supplementary Fig. 13 | Future change of EAJ variability in high summer. a**, Comparison of the SD in EAJ strength (m s$^{-1}$) in the present (1900–1999; blue bars) and future (2000–2099; orange bars) climate in 50 CMIP6 models with their multi-model mean. **b**, Same as **a**, but obtained from 50 members of CESM2 Large Ensemble (LENS2) with their ensemble mean. Error bars in the multi-model mean (ensemble mean) are SD of 10000 realizations (see "Bootstrap test" section in Methods).

**Supplementary Table 1 | Changes in Eurasian jet stream strength over three sub-regions in different datasets.** The jet stream strength indices used are described in "Indices" section of Materials and Methods. We use the epochal difference (P2 - P1) of SD to measure the change in amplitude, with the percent change of amplitude (difference relative to the SD during P1). Red and bold values denote they are significant at 0.05 confidence level.

| **Datasets** | **Change in SD** (m $s^{-1}$) | | | **Linear trend** (m $s^{-1}$ $decade^{-1}$) | | |
|---|---|---|---|---|---|---|
| | **EAJ** | **WAJ** | **WPJ** | **EAJ** | **WAJ** | **WPJ** |
| **ERA-5** | **+2.71 (+143.8%)** | -0.60 (-16.5%) | -0.69 (-16.5%) | -0.65 | -0.85 | 0.44 |
| **JRA-55** | **+2.77 (+145.4%)** | -0.59 (-16.5%) | -0.60 (-14.6%) | -0.61 | -0.70 | 0.43 |
| **ERA-I** | **+2.81 (+142.4%)** | -0.63 (-17.1%) | -0.69 (-16.7%) | -0.60 | -0.82 | 0.49 |
| **NCEP-NCAR** | **+2.87 (+136.0%)** | -0.32 (-9.4%) | -0.61 (-14.9%) | -0.89 | -0.65 | 0.35 |
| **NCEP/DOE** | **+2.91 (+139.9%)** | -0.42 (-11.8%) | -0.62 (-15.4%) | -0.90 | -0.78 | 0.34 |
| **MERRA-2** | **+2.74 (+140.0%)** | -0.68 (-18.0%) | -0.67 (-16.0%) | -0.56 | -0.98 | 0.50 |
| **CFSR** | **+2.61 (+124.0%)** | -0.49 (-13.8%) | -0.66 (-15.6%) | -0.64 | -0.71 | 0.51 |
| **Mean** | **+2.77 ± 0.10 （+138.8%）** | -0.53 ± 0.13 （-14.7%） | -0.65 ± 0.04 （-15.7%） | -0.78 ± 0.11 | -0.69 ± 0.14 | 0.43 ± 0.07 |

**Supplementary Table 2 | Change in EAJ interannual variability in different data.** The EAJ index used are described in "Indices" section of Methods. The inter-annual variability is determined by the SD (unit: m $s^{-1}$). The mean values are shown in red and bold.

| **Datasets** | **P1** | **P2** | **P2 (SCA removed)** |
|---|---|---|---|
| **ERA-5** | 1.88 | 4.59 | 2.50 |
| **JRA-55** | 1.90 | 4.67 | 2.47 |
| **ERA-I** | 1.98 | 4.79 | 2.50 |
| **NCEP-NCAR** | 2.11 | 4.98 | 2.52 |
| **NCEP/DOE** | 2.08 | 4.99 | 2.62 |
| **MERRA-2** | 1.96 | 4.70 | 2.58 |
| **CFSR** | 2.10 | 4.71 | 2.50 |
| **Mean** | **2.00 ± 0.10** | **4.77 ± 0.15** | **2.53 ± 0.05** |

**Supplementary Table 3 | Contribution of SCA on the enhanced variability of EAJ based on seven datasets. Contribution of SCA in SD of EAJ is calculated as the SD difference between the original SD and that after linearly removing the signal of SCA. The mean values are shown in red and bold.**

| **Datasets** | **Change in SD of EAJ from Table 1** (P2 – P1; m $s^{-1}$) | **Contribution of SCA in SD of EAJ** (m $s^{-1}$) | **Percentage** |
|---|---|---|---|
| **ERA-5** | 2.71 | 2.09 | 77.1% |
| **JRA-55** | 2.77 | 2.24 | 80.9% |
| **ERA-I** | 2.81 | 2.17 | 77.2% |
| **NCEP-NCAR** | 2.87 | 2.18 | 76.0% |
| **NCEP/DOE** | 2.91 | 2.36 | 81.1% |
| **MERRA-2** | 2.74 | 2.41 | 88.0% |
| **CFSR** | 2.61 | 2.29 | 87.7% |
| **Mean** | **2.77±0.10** | **2.248±0.11 (Rounded to 2.25±0.11)** | **81.1%±2.9%** |

**Supplementary Table 4 | Pattern correlations between the circulation modes shown in Supplementary Fig. 5.** Pattern correlations (see methods) are calculated for SCA pattern in the same region (25°N–85°N, 65°W–70°E) between that obtained from EAJ index and SCA index, at 200 hPa, 500 hPa and 850 hPa, respectively.

| Pattern Correlation | H200 | H500 | H850 |
|---|---|---|---|
| **Correlation mode** | 0.991 | 0.980 | 0.986 |
| **Regression mode** | 0.993 | 0.988 | 0.990 |

**Supplementary Table 5 | A list CMIP6 models used with corresponding institutions and resolutions.**

| No | Model Name | Institute | Resolution | No | Model Name | Institute | Resolution |
|---|---|---|---|---|---|---|---|
| **1** | ACCESS-CM2 | CSIRO-ARCCSS | 250 km | **26** | GFDL-CM4 | NOAA-GFDL | 100 km |
| **2** | ACCESS-ESM1-5 | CSIRO | 250 km | **27** | GFDL-ESM4 | NOAA-GFDL | 100 km |
| **3** | AWI-ESM-1-1-MR | AWI | 100 km | **28** | GISS-E2-1-G | NASA-GISS | 250 km |
| **4** | BCC-CSM2-MR | BCC | 100 km | **29** | GISS-E2-1-H | NASA-GISS | 250 km |
| **5** | CAMS-CSM1-0 | CAMS | 100 km | **30** | GISS-E2-2-G | NASA-GISS | 250 km |
| **6** | CAS-ESM2-0 | CAS | 100 km | **31** | HadGEM3-GC31-LL | MOHC NERC | 250 km |
| **7** | CanESM5 | CCCma | 500 km | **32** | HadGEM3-GC31-MM | MOHC | 100 km |
| **8** | CESM2 | NCAR | 100 km | **33** | IITM-ESM | CCCR-IITM | 250km |
| **9** | CESM2-FV2 | NCAR | 250 km | **34** | INM-CM4-8 | INM | 100 km |
| **10** | CESM2-WACCM | NCAR | 100 km | **35** | INM-CM5-0 | INM | 100 km |
| **11** | CIESM | THU | 100 km | **36** | IPSL-CM6A-LR | IPSL | 250 km |
| **12** | CMCC-CM2-SR5 | CMCC | 100 km | **37** | KACE-1-0-G | NIMS-KMA | 250 km |
| **13** | CMCC-ESM2 | CMCC | 100 km | **38** | KIOST-ESM | KIOST | 250 km |
| **14** | CNRM-CM6-1 | CNRM-CERFACS | 250 km | **39** | MCM-UA-1-0 | UA | 250 km |
| **15** | CNRM-ESM2-1 | CNRM-CERFACS | 250 km | **40** | MIROC-ES2L | MIROC | 500 km |
| **16** | E3SM-1-0 | E3SM-Project LLNL UCI UCSB | 100 km | **41** | MIROC-ES2H | MIROC | 250 km |
| **17** | E3SM-1-1 | E3SM-Project RUBISCO | 100 km | **42** | MIROC6 | MIROC | 250 km |
| **18** | E3SM-1-1-ECA | E3SM-Project | 100 km | **43** | MPI-ESM1-2-HR | MPI-M DWD DKRZ | 100 km |
| **19** | EC-Earth3 | EC-Earth-Consortium | 100 km | **44** | MPI-ESM1-2-LR | MPI-M AWI DKRZ DWD | 250 km |
| **20** | EC-Earth3-CC | EC-Earth-Consortium | 100 km | **45** | MRI-ESM2-0 | MRI | 100 km |
| **21** | EC-Earth3-Veg | EC-Earth-Consortium | 100 km | **46** | NESM3 | NUIST | 250 km |
| **22** | EC-Earth3-Veg-LR | EC-Earth-Consortium | 250 km | **47** | NorESM2-LM | NCC | 250 km |
| **23** | FGOALS-f3-L | CAS | 100 km | **48** | NorESM2-MM | NCC | 100 km |
| **24** | FGOALS-g3 | CAS | 100 km | **49** | TaiESM1 | AS-RCEC | 100 km |
| **25** | FIO-ESM-2-0 | FIO-QLNM | 100 km | **50** | UKESM1-0-LL | MOHC NERC NIMS-KMA NIWA | 100 km |
