# Supplementary material for: Atlantic origin of the increasing Asian westerly jet interannual variability
Source: Nat Commun. 2024 Mar 9;15:2155. doi: 10.1038/s41467-024-46543-x (PMC10925044; doi:10.1038/s41467-024-46543-x)
Supplement: Supplementary file 3 — Description of Additional Supplementary Files [file 41467_2024_46543_MOESM3_ESM.pdf]

Description of Additional Supplementary Files

File Name: Supplementary Code 1
Description: This zip file contains introduction, installation, usage and demo of using NCL (NCAR Command Language), as well as the reproduction instructions of figures in this study.
