# Supplementary figures and images for: Atlantic origin of the increasing Asian westerly jet interannual variability
Source: Nat Commun. 2024 Mar 9;15:2155. doi: 10.1038/s41467-024-46543-x (PMC10925044; doi:10.1038/s41467-024-46543-x)

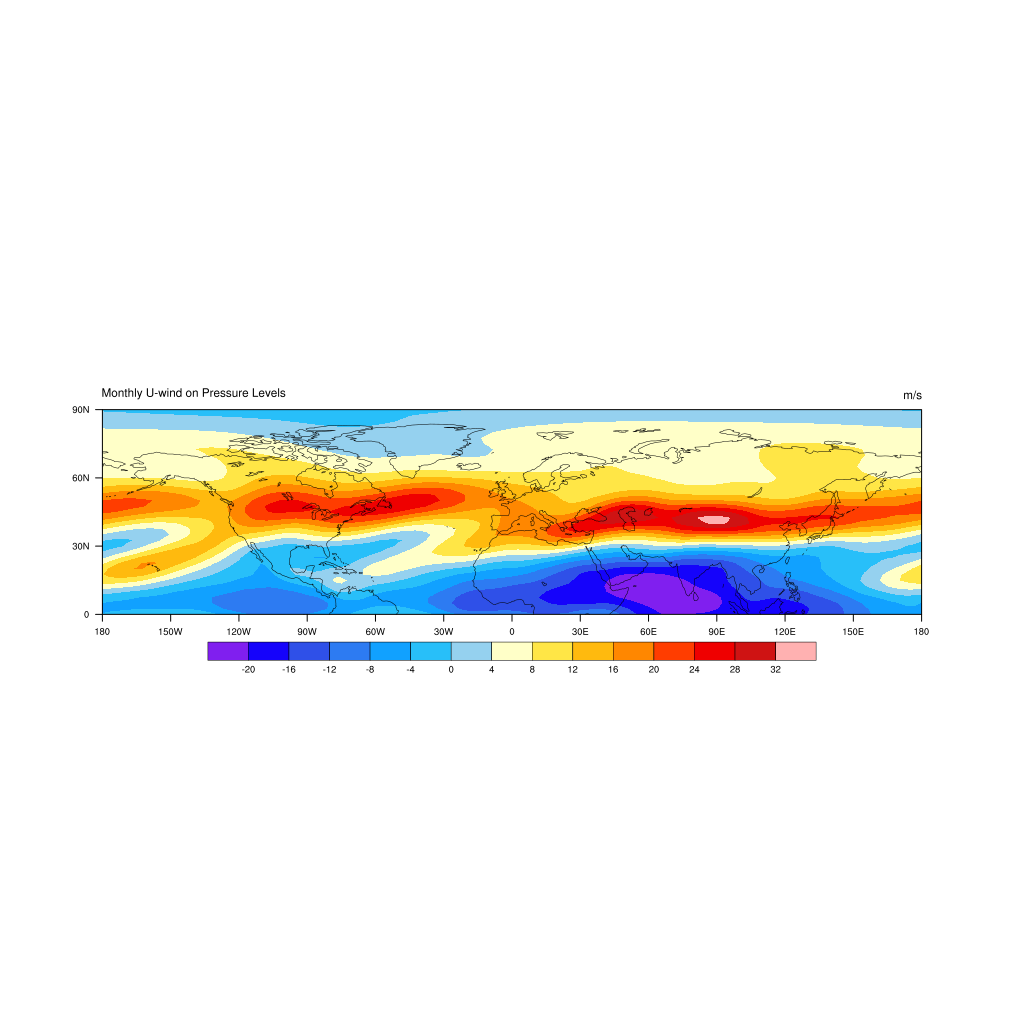

Supplement: Supplementary file 4 — Supplementary Code 1 [file 41467_2024_46543_MOESM4_ESM.zip › NC_soft/U200_Clim.png]
